# NK-like homeodomain proteins activate NOTCH3-signaling in leukemic T-cells

**DOI:** 10.1186/1471-2407-9-371

**Published:** 2009-10-19

**Authors:** Stefan Nagel, Letizia Venturini, Grzegorz K Przybylski, Piotr Grabarczyk, Corinna Meyer, Maren Kaufmann, Karin Battmer, Christian A Schmidt, Hans G Drexler, Michaela Scherr, Roderick AF MacLeod

**Affiliations:** 1 Dept. of Human and Animal Cell Lines, DSMZ - German Collection of Microorganisms and Cell Cultures, Inhoffenstr. 7B, 38124 Braunschweig, Germany; 2 Dept. of Hematology, Hemostasis, Oncology and Stem Cell Transplantation, Medical School Hannover, Carl-Neubergstr. 1, 30125 Hannover, Germany; 3 Institute of Human Genetics, Polish Academy of Sciences, Strzeszynska 32, 60-479 Poznan, Poland; 4 Dept. of Internal Medicine C, University of Greifswald, Sauerbruchstr., 17487 Greifswald, Germany

## Abstract

**Background:**

Homeodomain proteins control fundamental cellular processes in development and in cancer if deregulated. Three members of the NK-like subfamily of homeobox genes (NKLs), TLX1, TLX3 and NKX2-5, are implicated in T-cell acute lymphoblastic leukemia (T-ALL). They are activated by particular chromosomal aberrations. However, their precise function in leukemogenesis is still unclear. Here we screened further NKLs in 24 T-ALL cell lines and identified the common expression of MSX2. The subsequent aim of this study was to analyze the role of MSX2 in T-cell differentiation which may be disturbed by oncogenic NKLs.

**Methods:**

Specific gene activity was examined by quantitative real-time PCR, and globally by expression profiling. Proteins were analyzed by western blot, immuno-cytology and immuno-precipitation. For overexpression studies cell lines were transduced by lentiviruses.

**Results:**

Quantification of MSX2 mRNA in primary hematopoietic cells demonstrated higher levels in CD34+ stem cells as compared to peripheral blood cells and mature CD3+ T-cells. Furthermore, analysis of MSX2 expression levels in T-cell lines after treatment with core thymic factors confirmed their involvement in regulation. These results indicated that MSX2 represents an hematopoietic NKL family member which is downregulated during T-cell development and may functionally substituted by oncogenic NKLs. For functional analysis JURKAT cells were lentivirally transduced, overexpressing either MSX2 or oncogenic TLX1 and NKX2-5, respectively. These cells displayed transcriptional activation of NOTCH3-signaling, including NOTCH3 and HEY1 as analyzed by gene expression profiling and quantitative RT-PCR, and consistently attenuated sensitivity to gamma-secretase inhibitor as analyzed by MTT-assays. Furthermore, in addition to MSX2, both TLX1 and NKX2-5 proteins interacted with NOTCH-pathway repressors, SPEN/MINT/SHARP and TLE1/GRG1, representing a potential mechanism for (de)regulation. Finally, elevated expression of NOTCH3 and HEY1 was detected in primary TLX1/3 positive T-ALL cells corresponding to the cell line data.

**Conclusion:**

Identification and analysis of MSX2 in hematopoietic cells implicates a modulatory role via NOTCH3-signaling in early T-cell differentiation. Our data suggest that reduction of NOTCH3-signaling by physiological downregulation of MSX2 expression during T-cell development is abrogated by ectopic expression of oncogenic NKLs, substituting MSX2 function.

## Background

T-cells derive from early progenitor cells which in turn originate from CD34+ hematopoietic stem cells (HSC). After emigrating from the bone marrow, T-cells complete development in the thymus as thymocytes, passing several differentiation stages distinguished by the expression of surface proteins (e.g. CD3, CD4, CD8) and rearrangements of the T-cell receptor (TCR) genes [1]. Transcription factors LEF1, beta-Catenin and PU.1 and cytokines IL7, TGFbeta and BMP4 regulate thymocyte differentiation [2,3]. Furthermore, several signaling pathways are crucial for T-cell developmental processes, comprising TCR-, WNT- and NOTCH-pathways [4-6]. The last activates transcription factor CBF1/CSL/RBPJ which is associated with a repressor complex, mediating target gene silencing. This large complex contains several corepressor proteins, including SPEN/SHARP/MINT, TLE1/GRG1, CTBP and SKIP, and is localized in subnuclear aggregates [7-11]. Following ligand binding the transmembrane receptors NOTCH1 or NOTCH3, are proteolytically cleaved by gamma-secretase to release their intracellular domains, subsequently activating CBF1 by displacement of the repressor complex [6]. HES1/HRY and HEY1/HESR1/HRT1 are NOTCH activated target genes and members of the basic helix-loop-helix (bHLH) family of transcription factors. This family also includes their dimerization partners E12 and E47, representing fundamental regulators of lymphocyte differentiation [12]. Additional downstream effects of NOTCH comprise activation of the PI3K-pathway and of NFkB, enhancing survival of thymocytes [13,14].

Most oncogenes identified in T-cell acute leukemia (T-ALL) encode factors either regulating stage-specific thymocyte development, comprising NOTCH1, LMO2 and HOXA genes, or ectopically activated factors, including TAL1 and NK-like homeobox genes (NKLs) [15]. This gene family has been identified in *Drosophila*, comprising genes which essentially regulate fundamental steps in mesodermal and ectodermal differentiation [16-19]. Three NKL family members, TLX1/HOX11, TLX3/HOX11L2 and NKX2-5/CSX, act as master oncogenes in T-ALL. These genes are activated via chromosomal rearrangements and juxtaposed with either TCR genes or remote BCL11B enhancers displaying t(5;14)(q35;q32) [20-25]. Physiologically, TLX1 and NKX2-5 are expressed in developing spleen and, additionally, NKX2-5 in developing and adult heart [26,27]. Expression of TLX3 is restricted to cells of the peripheral nervous system [28]. Therefore, the leukemic actions of these genes might plausibly recapitulate their physiological activities as recently described for NKX2-5 [29]. Another related issue concerns whether similarities in oncogenic activity reflect kinship among homeobox genes.

Here we screened additional NKL leukemogenic candidates, thereby identifying common expression of MSX2 in T-cell lines. MSX2 is involved in organogenesis and differentiation of several tissues, including heart and the neural crest derivates teeth, hair follicles and bones [30]. Humans contain two MSX genes, MSX1 and MSX2. Both genes exhibit similar expression patterns and downstream effects [31,32]. Additionally, mice contain MSX3 which is not listed in human genome browsers. MSX2 interacts with several nuclear proteins, including corepressor proteins SPEN, TLE1, PIAS2/MIZ1 and H1E, and transcription factors DLX5 and RUNX2 [7,33-37]. Accordingly, MSX2 is involved in regulation of differentiation related genes, including Cyclin D1 (CCND1) and Osteocalcin [37,38] highlighting this ortholog as a fundamental regulator in development.

Here we identified MSX2 as physiological NKL involved in hematopoietic differentiation via regulation of NOTCH3-signaling. Our results indicate that this function of MSX2 might be replaced or modified by ectopic expression of oncogenic NKL family members in T-ALL.

## Methods

### Cell lines and treatments

Cell lines were supplied by the DSMZ (Braunschweig, Germany) except PER-117 provided by Ursula Kees, Perth, Australia. Cultivation was performed as described by Drexler [39]. Plasmid-DNA was introduced into cell lines by electroporation using the EPI-2500 impulse generator (Fischer, Heidelberg, Germany).

VSV.G-pseudotyped lentiviral particles were generated by calcium phosphate co-transfection of 293T cells and viral supernatants were concentrated as previously described [40]. Lentiviral transduction of cell lines Jurkat and MOLT-4 was performed twice with a multiplicity of infection (MOI) of approximately two. Transduced cells were sorted for EGFP-expression using Dako Cytomation MoFlo (Glostrup, Denmark).

For stimulation experiments the following reagents were used: cytokines IL7, TGFbeta and BMP4 (R&D Systems, Wiesbaden, Germany); antibodies anti-TGFBR2 (R&D Systems) and anti-CD3 (BD Biosciences, Heidelberg, Germany); chemical compounds Ionomycin, N-[N-(3,5-Difluorophenacetyl)-L-alanyl]-S-phenylglycine t-butyl ester (DAPT), 5-Aza-2'-deoxycytidine (AZA) and Rapamycin (Sigma-Aldrich, Taufkirchen, Germany), NFkB-inhibitor and Calphostin C (Calbiochem, Darmstadt, Germany).

### Primary cells

Peripheral blood cells (PBC), CD3+ and CD34+ cells were provided by the Medical School Hannover isolated from healthy donors, using the MACS system for cell preparations performed according to the manufacturer (Miltenyi Biotec, Bergisch Gladbach, Germany).

Twenty T-ALL samples were derived from patients which are included in the German ALL study group and provided by the University of Greifswald. The research was approved by an ethics committee. The samples were analyzed for TLX1/TLX3 expression by real-time PCR (see below). Three samples have been tested positive for TLX1 and seven for TLX3. Ten negative tested samples served as controls.

### RNA and cDNA

Total RNA was extracted from cells using TRIzol reagent (Invitrogen, Karlsruhe, Germany). cDNA was subsequently synthesized from 5 μg RNA by random priming, using Superscript II (Invitrogen).

### Polymerase chain reaction (PCR)

Reverse transcriptase (RT)-PCR was performed using taqpol (Qiagen) and thermocycler TGradient (Biometra, Göttingen, Germany). Oligonucleotides were obtained from MWG Eurofins (Martinsried, Germany). Their sequences are listed in Table 1. Quantitative expression analysis was performed by real-time PCR using the 7500 Real-time System, commercial buffer and primer sets (Applied Biosystems, Darmstadt, Germany). For normalization of expression levels we used TBP (Applied Biosystems). Copy number determination was performed using 50 ng genomic DNA per replicate. For normalization we used MEF2C as described recently [29]. Quantitative analysis were performed in triplicates and repeated twice.

**Table 1:** Oligonucleotides used for RT-PCR and ChIP

Gene	Acc.No	Forward primer (5'-3')	Reverse primer (5'-3')	PCR product (bp)
**HEX**	NM_002729	GCAAACCTCTACTCTGGAGC	TTCACTGGGCAAATCTTGCC	311

**HMX1**	NM_018942	AGGCGGCCTCAGTCCTGACA	TGCGGGAGAAGACTGTGCGC	263

**HMX2**	XM_370580	GCTTCACCATCCAGTCCATC	TTAAAGTCCGAGTGCGAAGG	295

**HMX3**	XM_291716	TGGCTTTCCCTCGCTTTGAG	TCCTCCAGAATGATCTCGTC	265

**MSX1**	NM_002448	AGAAGATGCGCTCGTCAAAG	ATCTTCAGCTTCTCCAGCTC	339

**MSX2**	XM_037646	AGATGGAGCGGCGTGGATGC	ACTCTGCACGCTCTGCAATGG	194

		GAATTCGAAGTCATGGCTTCTCCGTCC	TTGAATTCGGTGGTACATGCCATATCCC	811

**NKX1-1**	XM_926341	AATCTGACAGGAGCGATTGG	TGGAACCAGATCTTCACCTG	392

**NKX1-2**	XM_372331	TGGACCCACAGAAATTCACC	AACTTGTTCTCCAAGGCCAC	460

**NOTCH3**	NM_000435	CCGCACCCAGCCTATTATTG	AGAAGTGGGAGGATCGCTTG	219

		TAGACTGTCAGCTCCCTGAG	GCCCAGGAGTCTGAGGCTGC	156

**TEL**	NM_001987	AGGCCAATTGACAGCAACAC	TGCACATTATCCACGGATGG	272

### Protein analysis

Western blot analysis was performed as described previously [22]. Briefly, proteins obtained from cell lysates were transferred semi-dry onto nitrocellulose membranes (Bio-Rad, München, Germany) which were blocked with 5% bovine serum albumin dissolved in phosphate-buffered-saline buffer. Immunoprecipitation and immunocytology were performed as described previously [29]. The following antibodies were used: MSX2 (Affinity Bio Reagents, Golden, CO, USA); ERK1/2, NKX2-5, PML, TLX1 (Santa Cruz Biotechnology, Heidelberg, Germany); SPEN/SHARP (Bethyl Laboratories, Montgomery, TX, USA); TLE1 (Abnova, Taipei, Taiwan).

### Chromatin Immuno-Precipitation (ChIP)

ChIP analysis was performed using the ChIP Assay Kit obtained from Upstate (Lake Placid, NY). For immuno-precipitation we used antibody MSX2 (Affinity Bio Reagents), for nested PCR of NOTCH3 upstream sequences we used oligonucleotides as listed in Table 1. The procedure was performed as described previously [23].

### Fluorescence In Situ Hybridization (FISH)

FISH analysis was performed as described recently [41]. The following RP11-clones (obtained from the Sanger Centre, Cambridge, UK) were used as probes: 91K20, 704L16 (HEX); 117J13, 17I9 (HMX1); 487K11, 137E24 (HMX3); 1197E19, 117J13 (MSX1); 105I4, 54H11, 147G18 (MSX2). Additional fosmid clones, termed here "fosmid1" (G248P8229B6) and "fosmid2" (G248P8765G1) were used for detection of NKX2-5 and TLX3, respectively.

### Microscopy

For immunofluorescence microscopy of both chromosomes and cells we used an Axioskop 2 plus (Zeiss, Jena, Germany) and Cytovision 3.93 software (Applied Imaging, Newcastle, UK).

### Cloning procedures

MSX2 cDNA was obtained from Origene (Rockville, MD, USA), adjusted via PCR amplification and cloned in frame into pEGFP-N1 vector (Clontech, St-Germain-en-Laye, France). To construct the lentiviral plasmids S-MSX2-IEW, S-NKX2-5-IEW and S-TLX-1-IEW, the respective cDNA cassettes were blunt-end cloned into the blunted *Bam*HI site of the pHR'-SIN-SIEW-SnaBI vector, placing the cDNA fragment downstream of the SFFV promoter. RNA interference (i)-constructs directed against PU.1, LEF1 and beta-Catenin, respectively, have been described recently [23,42].

### Expression profiling

For quantification of gene expression via profiling we used DNA chips U133A Plus 2.0 obtained from Affymetrix, Buckinghamshire, UK. Chip-data analysis was performed as described recently [43]. Analysis of expression data was performed using online programs. For creation of heat maps we used CLUSTER version 2.11 and TREEVIEW version 1.60 http://rana.lbl.gov/EisenSoftware.htm. Those genes which displayed a minimal expression level of -2 and an up- or downregulation of at least 2-fold were selected for pathway analysis, using DAVID and KEGG provided by the National Center for Biotechnology Information (NCBI).

### MTT-assay

After diverse treatments for 16 h cell lines were subsequently prepared for standardized MTT (3-(4,5-dimethylthiazol-2-yl)-2,5-diphenyltetrazolium bromide; obtained from Sigma) assays. The absorbance was determined at 570 nm by an ELISA reader (Thermo Electron, Vantaa, Finland). Each approach was replicated (x6) and repeated (x2) with similar results.

## Results

### Screening of NK-like homeobox genes

Recently, we identified NKX2-5 as a novel, ectopically expressed, oncogenic homeobox gene in T-ALL cell lines and highlighted its relation to TLX1 and TLX3 for which an oncogenic role in T-ALL is well established [22]. These three genes are closely related members of the NKL family. A subsequent RT-PCR screen for additional family members expressed in T-ALL cell lines which included the human orthologous of *Drosophila *NK-genes yielded negative results [16,22].

Here, we extended that screen with respect to a comprehensive family definition [17], comprising additional 8 genes: HEX, HMX1/2/3, MSX1/2, NKX1-1/2. Expression of HMX2, NKX1-1 and NKX1-2 was not detected, relegating their putative role in T-cells or T-ALL, whereas HEX, HMX1/3 and MSX1/2 tested positive, albeit with substantial differences in their expression patterns (data not shown, Table 2). Since oncogenic expressions of TLX1, TLX3 and NKX2-5 are present in only few cell lines and coincide with chromosomal aberrations [22], we analyzed by FISH those NKL loci which showed restricted expression patterns, including HMX1, HMX3, HEX and MSX1. But all these genes displayed wild type configurations, lacking chromosomal abnormalities (data not shown, Table 2) to indicate oncogenic activation.

**Table 2:** RT-PCR and FISH analysis of selected NK-like homeobox genes

	MSX1	MSX2	HMX1	HMX2	HMX3	HEX	NKX1-1	NKX1-2
**ALL-SIL**	-	+ F	-	-	-	-	-	-

**CCRF-CEM**	+ F	+ F	-	-	-	+	-	-

**CML-T1**	-	+ F	-	-	+	-	-	-

**DND-41**	-	+ F	-	-	-	-	-	-

**HD-MAR**	-	- F	-	-	-	-	-	-

**HPB-ALL**	+ F	+ F	-	-	+ F	+	-	-

**H-SB2**	+	-	-	-	+	-	-	-

**HT-1**	-	+ F	-	-	-	-	-	-

**JURKAT**	+ F	+ F	-	-	+F	-	-	-

**KARPAS-45**	-	+ F	-	-	-	-	-	-

**KE-37**	+	+	-	-	-	-	-	-

**LOUCY**	+ F	+ F	-	-	-	+ F	-	-

**MHH-TALL2**	+ F	+ F	-	-	-	+ F	-	-

**MOLT-4**	+	+	-	-	-	+	-	-

**MOLT-14**	+ F	+	-	-	-	+	-	-

**MOLT-16**	- F	+ F	-	-	-	-	-	-

**P12-ICHIKAWA**	-	+	-	-	-	-	-	-

**PEER**	-	+ F	-	-	+ F	+ F	-	-

**PER-117**	nd F	+ F	nd	nd	nd	-	-	-

**PF-382**	-	+	-	-	+	+	-	-

**RPMI-8402**	-	+ F	-	-	-	+ F	-	-

**SUP-T1**	-	+	+ F	-	+ F	-	-	-

**TALL1**	-	+ F	-	-	-	-	-	-

**TALL-104**	+	+ F	-	-	-	-	-	-

**PBC**	-	+	nd	nd	nd	nd	nd	nd

**CD34+**	nd	+	nd	nd	nd	nd	nd	nd

positive expression (+), no expression (-), FISH analysis (F), not determined (nd)

**Figure 1:**
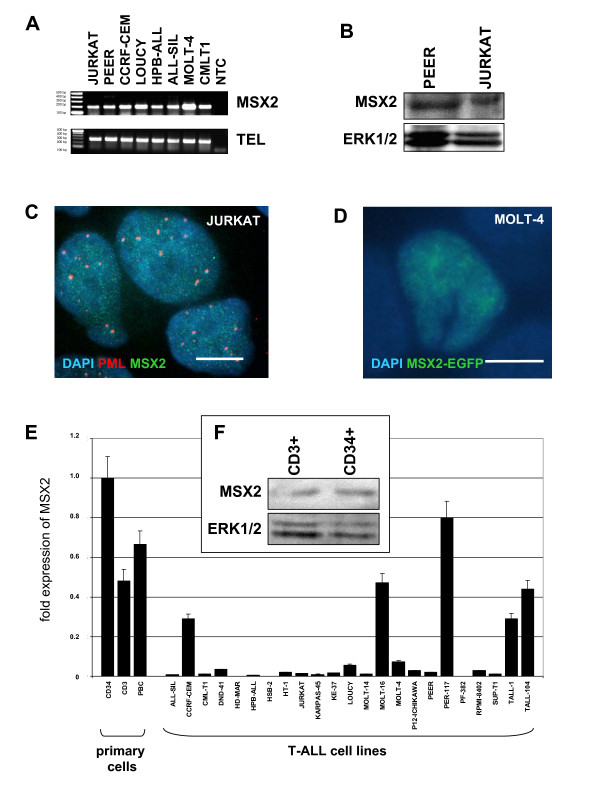
**Expression of MSX2**. **(A) **RT-PCR analysis indicates MSX2 expression in T-ALL cell lines. Expression of TEL serves as positive control. NTC: no template control. **(B) **Western blot analysis demonstrates MSX2 protein expression in PEER and JURKAT cells. Expression of ERK1/2 serves as loading control performed on a separate gel. **(C) **Immunocytological analysis in JURKAT cells. DAPI staining (blue) illustrates the nucleus. MSX2 staining (green) demonstrates a speckled distribution within the nucleus. The speckled pattern of PML staining (red) differs from that of MSX2. The scale bar represents 10 μm. **(D) **MOLT-4 cells were transfected by electroporation with expression construct pMSX2-EGFP. The fusion protein MSX2-EGFP (green) shows a speckled distribution within the nucleus (blue), resembling that of endogenous MSX2. **(E) **Quantitative expression analysis of MSX2 by real-time PCR in primary hematopoietic cells (CD34+, CD3+, PBC) and 23 T-ALL cell lines revealed striking differences. MSX2 expression levels are shown in relation to that of CD34+ cells which was set to 1. Expression of TBP served as endogenous control. Bars show standard deviations. **(F) **Western blot analysis demonstrates MSX2 protein expression in primary CD34+ and CD3+ cells. ERK1/2 serves as loading control, indicating higher amounts of MSX2 protein in CD34+ cells.

MSX2 showed the most widespread expression pattern as detected in 22/24 (92%) of the analyzed cell lines (Figure 1A, Table 2) and, therefore, may represent a promising candidate physiological NKL in T-cells. The physiological function of MSX2 might be related to or modified by aberrant NKLs and its investigation may contribute to understanding their oncogenic role(s) in T-ALL. To address this issue we analyzed expression, regulation and function of MSX2 in T-cells.

### Expression of MSX2 in hematopoietic cells

MSX2 protein expression was confirmed by western blot analysis in two T-ALL cell lines, PEER and JURKAT (Figure 1B). The same antibody used for immunofluorescence microscopy of JURKAT cells revealed a speckled pattern within the nucleus distinct from that of PML protein (Figure 1C). An expression construct, fusing MSX2 and green fluorescence protein (pMSX2-EGFP) was electroporated into MOLT-4 cells and yielded a similar nuclear distribution of the fusion protein (Figure 1D). These results suggest that MSX2 protein possesses an intrinsic capacity for subcellular localization in T-cells which may be significant for regulation of target genes.

For expression analysis of primary cells we quantified MSX2 mRNA levels in CD34+ HSCs, CD3+ T-cells and peripheral blood cells (PBCs) by real-time PCR. The data showed circa 2-fold higher expression levels in CD34+ HSCs compared to CD3+ T-cells and PBCs (Figure 1E). This quantitative difference in MSX2 expression was also detected at the protein level as analyzed by western blot (Figure 1F). These results demonstrate physiological expression of MSX2 in hematopoietic cells and indicate transcriptional downregulation during T-cell development.

### Regulation of MSX2 expression in T-cells

**Figure 2:**
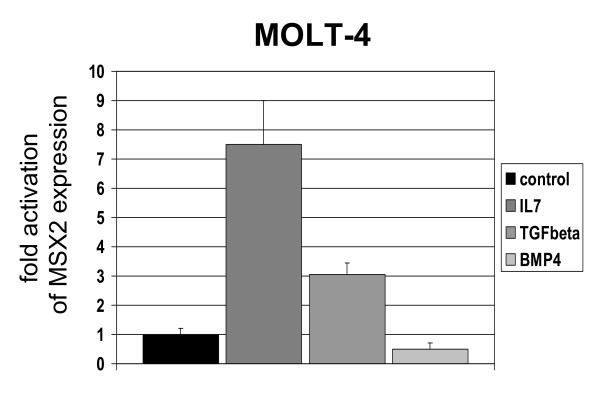
**Regulation of MSX2 expression**. MOLT-4 cells were treated for 16 h with 20 ng/ml IL7, TGFbeta, or BMP4, respectively. Subsequent expression of MSX2 was quantified by real-time PCR. In comparison to untreated control cells (set to 1) the level of MSX2 changed about 7-, 3- or 0.5-fold, indicating regulation by these thymic factors. Standard deviations are indicated by bars.

To examine the regulation of MSX2 transcription in T-cells we analyzed several factors relevant to thymic T-cell differentiation, including IL7, BMP4, TGFbeta, PU.1, LEF1, beta-Catenin, CD3, calcium and NOTCH-signaling in the cell lines JURKAT and MOLT-4. MSX2 mRNA expression levels rose 7-fold and 3-fold after treatment with IL7 and TGFbeta, respectively, and decreased 0.5-fold with BMP4 when analyzed by real-time PCR (Figure 2, Table 3). Consistently, inhibition of TGFbeta receptor (TGFBR2) by an appropriate antibody decreased MSX2 expression 0.6-fold (Table 3). The effects of transcription factors PU.1, LEF1 and beta-Catenin were analyzed by their RNAi-mediated knockdown in MOLT-4 cells. Subsequent expression analysis indicated activation of MSX2 transcription by PU.1 and beta-Catenin, respectively, and inhibition by LEF1 (Table 3). Treatment of cells with the calcium ionophore Ionomycin or an activating CD3-antibody resulted in decreased MSX2 expression, indicating inhibition by TCR-signaling (Table 3). Using gamma-secretase inhibitor DAPT, we detected a slight inhibition via NOTCH-signaling, showing 1.7-fold activation of MSX2 expression (Table 3). Taken together, these results demonstrate that MSX2 transcription is regulated by core thymic factors which are involved in T-cell differentiation, supporting the view of MSX2 as a developmentally regulated physiological NKL in thymocytes.

**Table 3:** Fold expression of MSX2 mRNA after treatment with core thymic factors as analyzed by real-time PCR

Factor	JURKAT	MOLT-4
**IL7**	~ +/- 0.3	x7.5 +/- 1.5

**BMP4**	x0.5 +/- 0.2	x0.4 +/- 0.2

**TGFbeta**	nd	x3.0 +/- 0.3

**anti-TGFBR2**	~ +/- 0.2	x0.6 +/-0.2

		

**sh-PU.1**	nd	x0.7 +/- 0.1

**sh-LEF1**	nd	x3.0 +/- 1.5

**sh-betaCatenin**	nd	x0.01 +/- 0.1

		

**anti-CD3**	x0.5 +/-0.2	nd

**Ionomycin**	x0.5 +/-0.2	x0.1 +/-0.2

		

**DAPT**	x1.6 +/-0.2	

standard deviations (+/-), not determined (nd), nochange in expression (~)

**Figure 3:**
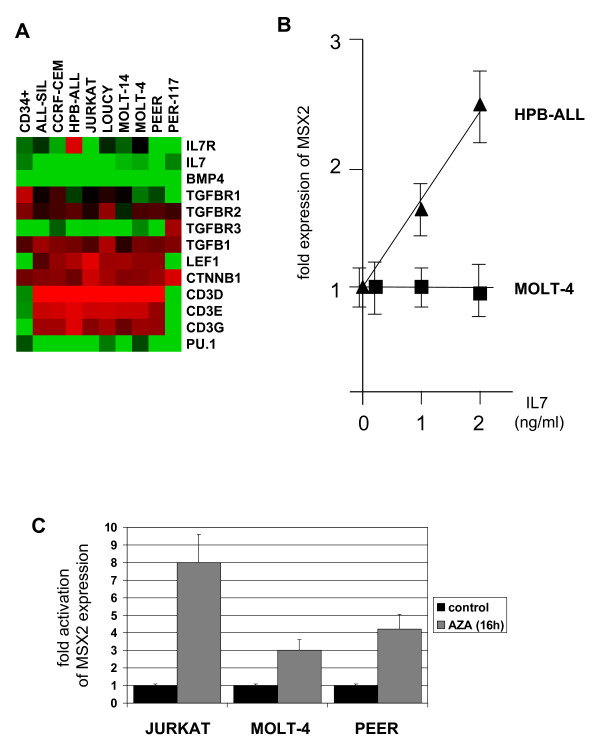
**Differential regulation of MSX2**. **(A) **Nine cell lines in addition to CD34+ primary HSCs were analyzed by expression profiling. The heat map shows expression levels of genes involved in regulation of MSX2 in T-cell lines. Red indicates high, green low and black intermediate expression levels. Of note, HPB-ALL expresses high levels of IL7R; PER-117 expresses high levels of TGFBR3 and low levels of LEF1, CD3D, CD3E and CD3G. **(B) **HPB-ALL and MOLT-4 cells were treated for 16 h with low amounts of IL7. Subsequent analysis of MSX2 expression by real-time PCR indicates increasing levels in HPB-ALL (expresses high IL7R levels) but no change in MOLT-4 (expresses low IL7R levels). Bars show standard deviations. **(C) **T-cell lines JURKAT, MOLT-4 and PEER were treated with 500 nM 5-Aza-2'-deoxycytidine for 24 h. Subsequent real-time PCR analysis indicates about 8-, 3-, and 4-fold increased levels of MSX2 expression, respectively.

The expression levels of MSX2 mRNA in T-ALL cell lines varied substantially from very low, (e.g. HPB-ALL), to high, (e.g. PER-117) (Figure 1E). Therefore, expressions of core thymic factors and their receptors shown to regulate MSX2 transcription were analyzed by expression profiling in 9 T-ALL cell lines in addition to CD34+ primary HSCs (Figure 3A). These data suggested that high expression levels of TGFbeta receptor (TGFBR3) and low expression levels of LEF1 and CD3 may, respectively, contribute to elevated MSX2 expression in PER-117. Furthermore, all cells expressed low levels of IL7 and IL7 receptor except HPB-ALL which showed high IL7 receptor levels (Figure 3A). This fact was utilized to analyze the effect of IL7-signaling on MSX2 expression in more detail by treating HPB-ALL and MOLT-4 with very low amounts of IL7. The level of MSX2 mRNA increased strongly in HPB-ALL but remained constant in MOLT-4, highlighting the potential of IL7-signaling for MSX2 expression (Figure 3B). Thus these results showed that differences in signaling of core thymic factors contribute to varied expression levels of MSX2.

The MSX2 locus contains five CpG islands http://www.genome.ucsc.edu/, suggesting epigenetic regulation via genomic methylation. Accordingly, treatment of T-ALL cell lines with demethylating agents resulted in increased levels of MSX2 expression, ranging from 3 to 8-fold (Figure 3C), supporting a role for DNA methylation in transcriptional regulation.

**Figure 4:**
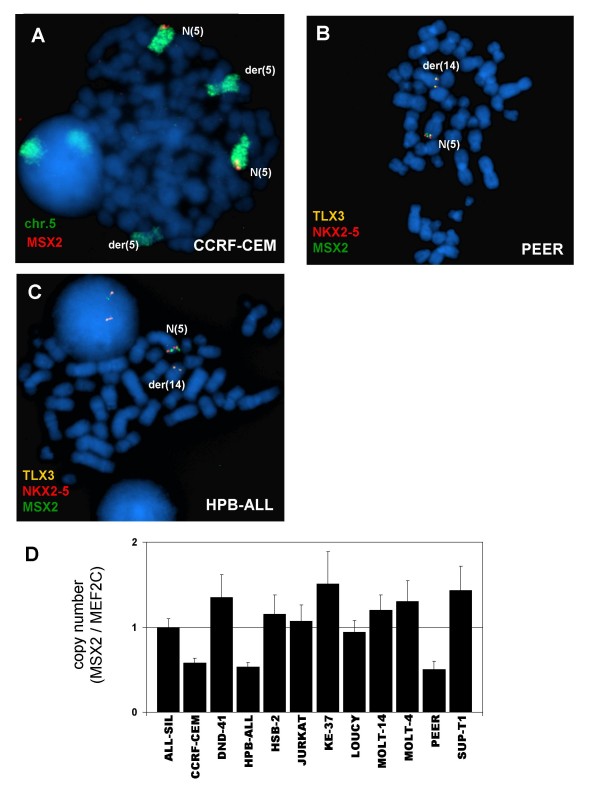
**Copy number analysis of MSX2**. **(A) **FISH analysis of tetraploid CCRF-CEM cells, using a painting probe for chromosome 5 (green) and RP11-54H11 probe for MSX2 (red). Of note, one allele of MSX2 (located at 5q35) is deleted on der(5). FISH analysis of PEER **(B) **and HPB-ALL **(C)**, using probes for MSX2 (54H11, green), NKX2-5 (fosmid1, red) and TLX3 (fosmid2, orange). Data indicate deletion of MSX2 with concomitant rearrangement of NKX2-5 (PEER) or TLX3 (HPB-ALL). **(D) **Quantitative analysis of MSX2 copy numbers was performed by genomic real-time PCR in 12 selected T-cell lines. The MEF2C locus was used as endogenous control as described previously [29]. Of note, cell lines CCRF-CEM, HPB-ALL and PEER demonstrate bisected quantities, indicating deletion of one allele of MSX2.

To check if the locus of MSX2 at chromosome 5q35 displays rearrangements which may influence expression we analyzed several T-ALL cell lines with varying levels of MSX2 mRNA by FISH (Table 2). While neither translocations nor amplifications were detected, in 3 out of 17 cell lines FISH indicated deletion of one MSX2 allele (Figure 4A-C). Subsequent analysis of genomic DNA by quantitative real-time PCR confirmed deletion of MSX2 alleles in cell lines CCRF-CEM, HPB-ALL and PEER (Figure 4D). However, the copy number in these cell lines did not correlate with reduced MSX2 mRNA levels as demonstrated for CCRF-CEM (Figure 1E), indicating that MSX2 is not a target of this chromosomal aberration. Taken together, we have identified several factors which may influence transcription of MSX2 in T-cells, including signaling by core thymic factors and genomic methylation while excluding gene dosage.

### Functional analysis of MSX2

**Figure 5:**
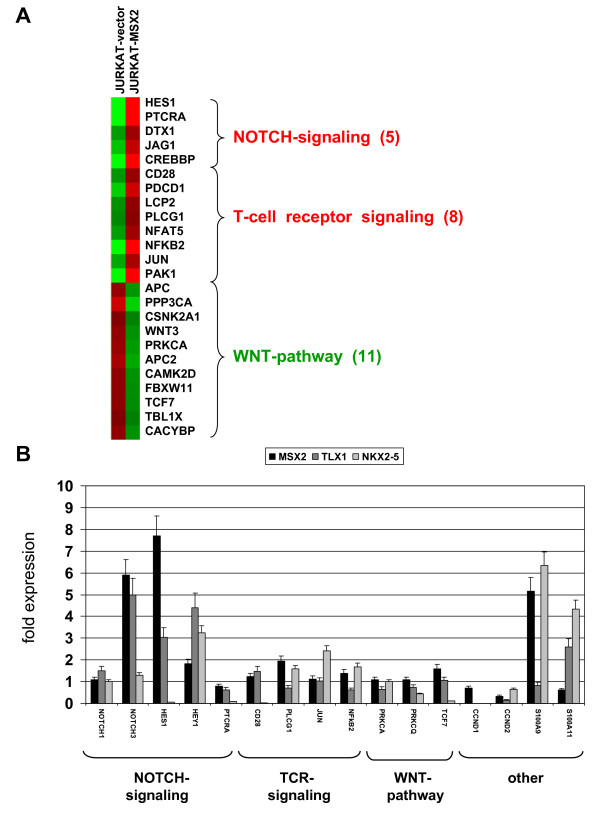
**Functional analysis of MSX2**. **(A) **Heat-map of selected pathway-genes. Expression data obtained by profiling of JURKAT cells transduced with empty vector and MSX2, respectively, were transformed into a heat-map, demonstrating differential gene activities. **(B) **Quantitative real-time PCR expression analysis of 16 candidate target genes was performed in JURKAT cells transduced with MSX2, TLX1 or NKX2-5, respectively, in comparison to vector controls. Expression of TBP served as endogenous control.

**Figure 6:**
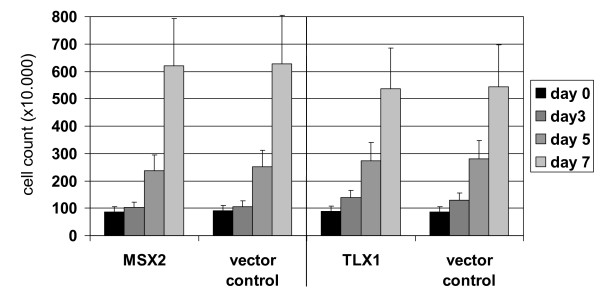
**MSX2 and T-cell proliferation**. Proliferation analysis of modified T-cell line. JURKAT cells tranduced with empty vector, MSX2 or TLX1, respectively, were counted during a period of one week. Cell counts indicated no significant differences in proliferation.

Combined data on expression and regulation analysis attest that MSX2 is a physiological NKL in hematopoietic cells downregulated in T-lymphocytes. To examine the function of MSX2 in T-cells we lentivirally transduced cell line JURKAT for overexpression. For identification of genes regulated downstream of MSX2 we performed expression profiling of JURKAT-MSX2 in comparison to JURKAT-vector cells. Using the online DAVID/KEGG software, we identified upregulation of NOTCH- and TCR-pathway genes and downregulation of WNT-pathway genes (Figure 5A). Furthermore, among the top 100 upregulated genes expression of S100A9 was striking. S100A9 belongs to a family of clustered genes, including S100A11 preferentially expressed in T-cells. Next we quantified expression of 16 potential target genes by real-time PCR: NOTCH1, HES1, PTCRA, CD28, PLCG1, JUN, NFkB2, PRKCA, PRKCQ, TCF7, S100A9 and S100A11 in addition to reported targets of MSX1, including NOTCH3, HEY1 and CCND1/2 (Figure 5B) [38,44]. These data confirmed a significant activatory role for MSX2 in the expression of NOTCH-pathway genes and S100A9 whereas the influence on TCR- and WNT-signaling genes was not conclusive. MSX2 activated expression of NOTCH-target genes HES1 and HEY1 in addition to NOTCH3, while excluding NOTCH1 indicated stimulation of the NOTCH3-pathway. Sequence analysis of the NOTCH3 promoter region identified a consensus sequence for MSX2 binding located far upstream at about -28 kbp [45]. But subsequent ChIP analysis in JURKAT-vector/MSX2 cells failed to detect direct binding of MSX2 protein at this particular site (data not shown). In contrast to previous reports [38], MSX2 mediated downregulation of cyclin D genes (CCND1 and CCND2), which suggested repressive involvement in proliferation. However, subsequent proliferation analysis of JURKAT-MSX2 and JURKAT-TLX1 showed no differences in growth after 7 days of cultivation (Figure 6).

These results indicate a role of MSX2 in early T-cell differentiation and mark the NOTCH3-pathway as a potential target of oncogenic NKLs. To check their (dys)regulating effects we transduced JURKAT with TLX1 and NKX2-5 and subsequently determined expression levels of candidate target genes (Figure 5B). Both proteins, TLX1 and NKX2-5, resemble MSX2 in activating NOTCH3 and HEY1 when overexpressed in JURKAT cells. HES1 was activated by MSX2 and TLX1 and repressed by NKX2-5. S100A9 was activated by MSX2 and NKX2-5 and S100A11 by TLX1 and NKX2-5. Therefore, we identified NOTCH3-pathway and S100A genes as targets of both physiological and oncogenic NK-like homeodomain proteins in T-cells.

**Figure 7:**
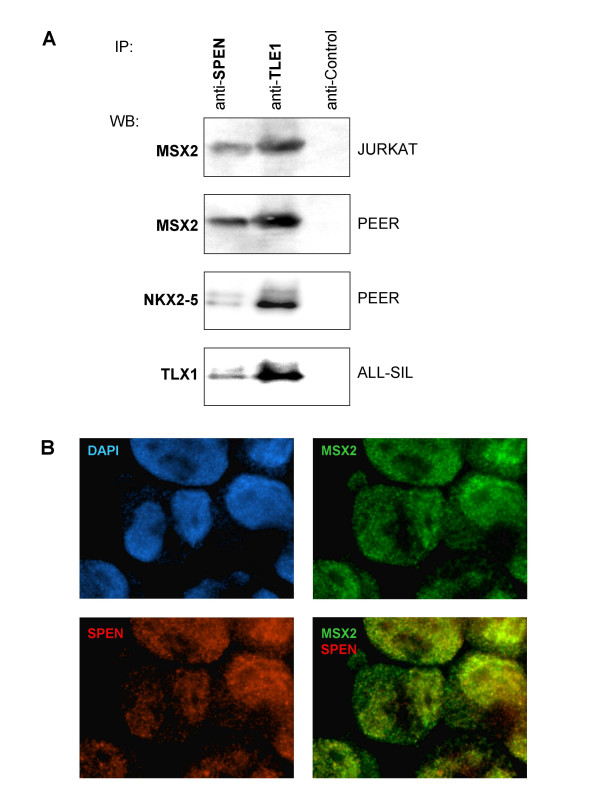
**Protein interactions of NKL proteins**. **(A) **Immunoprecipitation analysis (IP) has been performed for SPEN and TLE1, respectively, in JURKAT, PEER and ALL-SIL. Subsequent Western blot analysis (WB) indicates interaction with MSX2, NKX2-5 and TLX1, respectively. **(B) **Immunocytological analysis of MSX2 and SPEN was performed in JURKAT cells. DAPI staining (blue) illustrates the nucleus. Both, MSX2 (green) and SPEN (red) demonstrate a speckled distribution within the nucleus and large colocalizations (yellow).

Consistent with involvement in NOTCH3-pathway activation, MSX2 has been reported to interact with SPEN and TLE1 in two other cell types, namely fibroblasts and kidney, respectively [33,37]. Both are corepressors implicated in regulation of NOTCH-signaling. We confirmed these interactions in T-cells by immunoprecipitation (Figure 7A) and by immunofluorescence microscopy (Figure 7B), showing colocalization of MSX2 and SPEN in subnuclear aggregates. These results suggest that MSX2 might modulate the activity of the CBF1 repressor complex, containing corepressors SPEN and TLE1, in T-cells. Moreover, both corepressor proteins interacted with TLX1 and NKX2-5 (Figure 7A). Therefore, these protein interactions may represent a potential oncogenic mechanism, resulting in dysregulation of CBF1.

**Figure 8:**
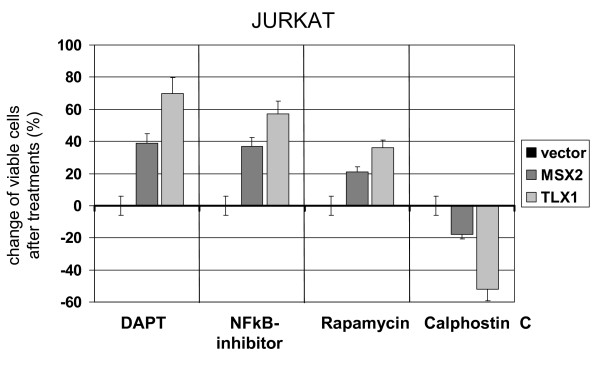
**Survival of NKL expressing T-ALL cells**. JURKAT cells transduced with MSX2, TLX1 or vector control, respectively, were treated for 16 h with pharmacological inhibitors of NOTCH signaling, NFkB activity, PI3K-signaling or PKC. Subsequent analysis of cell viability by MTT assay indicates differences in sensitivities. Values of JURKAT-vector cells were used as control. Bars show standard deviations.

To check the impact of physiological MSX2 and oncogenic TLX1 on cell survival we treated transduced JURKAT cells with gamma-secretase-inhibitor DAPT, NFkB-inhibitor, PI3K-pathway-inhibitor Rapamycin as well as Calphostin C, which triggers calcium-dependent apoptosis in ALL cells [46]. Subsequent analysis of cell viability by MTT assay indicated for both MSX2 and TLX1 transduced cells reduced sensitivities to DAPT, NFkB-inhibitor and Rapamycin, but enhanced sensitivity to Calphostin C (Figure 8).

**Figure 9:**
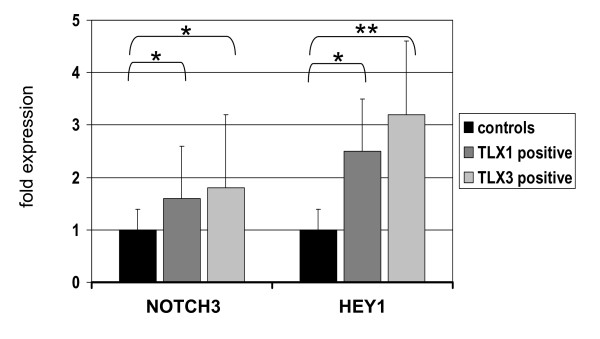
**Expression analysis of primary T-ALL cells**. Quantitative real-time expression analysis of NOTCH3 and HEY1, was performed in primary T-ALL cells by real-time PCR. Expression of TBP served as endogenous control. The figure indicates mean expression levels of analyzed probes. Bars show standard deviations. The significance of these data was calculated using the t-test with p = 0.03 (*) and p = 0.01 (**).

Finally, expression analysis of primary T-ALL cells demonstrated elevated levels of both NOTCH3 and HEY1 in TLX1 and TLX3 positive samples (Figure 9), confirming the stimulation of NOTCH3-signaling by oncogenic NKLs in corresponding patients.

## Discussion

We have identified expression of NKL MSX2 in T-ALL cell lines as well as in primary HSCs and T-cells. MSX2 expression in cell lines was regulated by core thymic factors, suggesting that MSX2 is a physiological NKL in hematopoietic cells, notably in T-cells/thymocytes. Analysis of transduced JURKAT cells overexpressing either MSX2 or oncogenic TLX1 and NKX2-5 identified activation of NOTCH3-signaling, including bHLH target gene HEY1. Expression levels of NOTCH3 and HEY1 were elevated in primary TLX1/3 positive T-ALL cells, underpinning the cell line data.

MSX2 expression has been described in stem cells, progenitor cells and derivates of neural crest cells, indicating a role in cell differentiation [47]. Core stem cell transcription factors directly regulate expression of MSX2, demonstrating a prominent function of this homeobox gene in the regulatory network of undifferentiated cells [48]. MSX2 knockout mice exhibit several abnormalities in tissues derived from neural crest cells but none in the hematopoietic system [49-51]. However, homeostatic compensations among MSX genes as described for some tissues may explain the absence of any hematopoietic phenotype hitherto [51]. Of note, expression of oncogenic NKLs have also been reported in neural crest derivates, including TLX1 in teeth, TLX3 in dorsal root ganglia and NKX2-5 in heart and teeth which may suggest correlations in the regulation of differentiation processes [28,52,53].

In T-cell lines we analyzed several core thymic factors which differently influenced MSX2 expression. The activity of these factors correlates with particular stages of thymocyte development, showing IL7 and TGFbeta activity in early stages, and BMP4, LEF1 and TCR-signaling in later stages of thymocyte differentiation [2,3]. Combined data, concerning both higher MSX2 expression levels in primary HSCs as compared to CD3+ T-cells and particular MSX2 regulation, activating by early and suppressing by late core thymic factors, indicate physiological downregulation of MSX2 during T-cell development. Accordingly, PER-117 which expresses high MSX2 levels represents a very immature T-cell line corresponding to stage I thymocytes [54]. Additionally, our data indicated an impact of DNA methylation on MSX2 expression. However, whether this mechanism represents the physiological situation is unclear.

Interestingly, we detected chromosomal deletions of MSX2 loci at 5q35 in three T-ALL cell lines which contain t(5;14)(q35;q32) rearrangements, targeting TLX3 or NKX2-5 (also located at 5q35 centromeric of MSX2), respectively [22]. Cooperation of genetic defects t(5;14)(q35;q32) and del(5)(q35) has been reported in primary T-ALL samples [55], suggesting that MSX2 may be a target of del(5)(q35) in this entity. But the poor correlation with expression data indicated that MSX2 is not a plausible target of this chromosomal aberration. We suggest that TLX3 and NKX2-5 are main targets and deletion of MSX2 is incidental because of its physiological downregulation and functional substitution by those oncogenic NKLs.

NOTCH3 has a major impact on T-cell development, by regulating differentiation and survival of thymocytes [56,57]. Here we identified MSX2 mediated activation of the NOTCH3-pathway in T-cells reminiscent of MSX1 in neuroblastoma [44]. We failed in detecting direct binding of MSX2 protein to a far upstream located potential binding site [45]. However, other studies demonstrate indirect impacts of MSX2 in target gene activities [37,58,59]. Furthermore, MSX2 colocalized with SPEN in JURKAT cells but not with PML and interacted with SPEN and TLE1, both components of the CBF1 associated repressor complex. This complex has been localized to subnuclear aggregates which differ from those containing PML protein and which comprise CBF1, SPEN, TLE1, SKIP and activated NOTCH [7-11,60]. Therefore, our results reveal the impact of MSX2 on CBF1 target gene regulation. Since bHLH genes HES1 and HEY1 are regulated by CBF1 and NOTCH3 [13,61], we conclude that MSX2 regulates HES1 and HEY1 via both CBF1 repressor complex modulation and activation of NOTCH3 expression. The subnuclear distribution of these aggregates seems to be important for their activity. Noteworthy in this context, MSX2 has been described to interact with SUMO-ligase PIAS2 [62]. PIAS proteins may regulate distribution and activity of NK-like homeodomain proteins via SUMOylation as recently described for MSX1 and NKX2-5 [63,64].

Additional downstream effects of NOTCH1/3-signaling comprise activation of NFkB and of PI3K-pathway, enhancing survival of thymocytes [13,65-67]. Accordingly, both activities were elevated in JURKAT cells overexpressing MSX2 or TLX1, as demonstrated by reduced sensitivities to inhibitors of gamma-secretase, NFkB and PI3K-signaling.

Oncogenic NKLs TLX1 and NKX2-5 resemble MSX2 in interacting with SPEN and TLE1. Accordingly, a direct interaction between TLX1 and TLE1 has been shown recently [68]. In addition, both TLX1 and NKX2-5 activate the NOTCH3 target gene HEY1. These results suggest that both proteins substitute MSX2 in modulating the CBF1 associated repressor complex. In JURKAT cells overexpressing oncogenic NKLs we observed a shift in target gene activity from HES1 to HEY1, suggesting differences in their mode of interaction with SPEN. However, both HES1 and HEY1 are bHLH proteins and involved in inhibition of differentiation via interaction with E2A proteins E12 and E47 [12]. Therefore, our results imply that NK-like homeodomain proteins activate CBF1 target genes, thereby inhibiting T-cell differentiation.

Furthermore, we have identified S100A genes as targets of NKLs. S100A9/11 are calcium-binding proteins which inhibit PKC-mediated phosphorylation of bHLH proteins and activate apoptosis in ALL cells [69,70]. Our data suggest a correlation between S100A expression and sensitivity for Calphostin C. Mechanistically, Calphostin C may enhance apoptosis either via elevation of intracellular calcium levels or via inhibition of PKC, modulating activities of bHLH proteins [46,69,71]. However, this promising apoptotic effect merits further examination.

Recently, we have identified MEF2C as specific oncogenic target of NKX2-5 in T-ALL cells, reflecting the physiological function of NKX2-5 in the heart [29]. Here, we identified NOTCH3-signaling activated by both physiological MSX2 and oncogenic TLX/NKX2-5 in T-ALL cells. Thus, both modes of leukemic action may be displayed by NKLs in T-ALL, ectopic activations related to their physiological origin and dysregulations due to structural similarities to physiological members of this homeobox gene family.

## Conclusion

The identification of MSX2 as a physiological NKL in hematopoietic cells and its involvement in NOTCH3-signaling further implicates this pathway in crosstalk between physiological and oncogenic homeobox signaling in T-ALL.

## Competing interests

The authors declare that they have no competing interests.

## Authors' contributions

SN designed the research and wrote the paper, LV, KB and MS performed lentiviral gene transfer, GKP, PG and CAS contributed patient samples, CM and MK performed the labwork, HGD supported the labwork, RAFM designed the research.

## Pre-publication history

The pre-publication history for this paper can be accessed here:

http://www.biomedcentral.com/1471-2407/9/371/prepub
